# Development of thermo-photo sensitive genic male sterile lines in wheat using doubled haploid breeding

**DOI:** 10.1186/s12870-020-02458-5

**Published:** 2020-06-01

**Authors:** Hongsheng Li, Shaoxiang Li, Sedhom Abdelkhalik, Armaghan Shahzad, Jian Gu, Zhonghui Yang, Mingliang Ding, Kun Liu, Hong Zhao, Mujun Yang

**Affiliations:** 1 Institute of Food Crops, Yunnan Academy of Agricultural Sciences / Yunnan Branch of National Wheat Improvement Center, Kunming, Yunnan 650205 P. R. China; 2 Filed Crops Research Institute, Agricultural Research Center, Kafr El-Shaikh, 33717 Egypt; 3 National Institute for Genomics and Advanced Biotechnology, National Agricultural Research Centre, Islamabad, 45500 Pakistan

**Keywords:** Hybrid wheat, Two lines system, Thermo-photo sensitive genic male sterility, Doubled haploid, Wheat × maize

## Abstract

**Background:**

Two-line hybrid wheat system using thermo-photo sensitive genic male sterility (TPSGMS) is currently the most promising approach for wheat heterosis utilization in China. However, during past 20 years only few TPSGMS lines were developed in hybrid wheat breeding, which has been the main limiting factor to create heterotic hybrids. Application of doubled haploid (DH) breeding provides a useful strategy to efficiently develop practically usable TPSGMS lines.

**Results:**

F_1_s and selected F_2_ and F_3_ sterile plants of eight crosses made from two commercial TPSGMS lines were used to produce DH lines. We developed a total of 24 elite DH sterile lines with stable sterility, good outcrossing and yield potential, resistance to yellow rust and powdery mildew, as well as desirable plant height (50–60 cm). These DH lines were developed within 4 years through at least 1 year of evaluation. The stability of male sterility was confirmed for most (20/24) of these elite DH sterile lines by multiple tests in two or 3 years. These lines are expected to be used in hybrid wheat breeding. The percentage of elite lines developed from the tested DH lines produced from filial generations was in the order of F_2_ > F_3_ > F_1_.

**Conclusions:**

We demonstrate that coupling DH techniques with conventional breeding is an efficient strategy for accelerating the development of more practical wheat TPSGMS lines. Generation of DHs from F_2_ generation appeared to be the better choice considering the balance of shortening breeding time and overall breeding efficiency.

## Background

Wheat provides about 20% of the world’s nutrition supply [1]. Heterosis utilization in wheat is one of the most promising ways for increasing yield potential and stability, which has importance for increasing the productivity of wheat to meet the growing demand in the world [2–4]. However, developing hybrids with high level of heterosis and producing hybrid seeds with low cost remain challenges in hybrid wheat breeding and its commercial application [1, 4].

Unlike hybrid wheat systems based on cytoplasmic male sterility (CMS) [5] and photoperiod-sensitive cytoplasmic male sterility (PCMS) [6], the two-line hybrid wheat system using thermo-photo sensitive genic male sterility (TPSGMS) is a new methodology of wheat heterosis utilization in China. The TPSGMS line is characterized as being sterile under low-temperature and short-day for hybrid seed production, and fertile under high-temperature and long-day for self propagation. Therefore, this system does not need a sterility ‘maintainer’ line and makes hybrid seed production easier [7–9]. From 2002 to 2018, 20 hybrid wheat varieties were released in China [10], 14 of them were developed using TPSGMS-based two-line system with yield increase of 10–15%, especially in marginal lands [10–12]. Meanwhile, encouraging multi-location evaluations have been conducted and observed in Vietnam, where “Yunza” hybrid varieties performed much better than local inbred cultivars in yield, drought tolerance and fertilizer input [13]. Nevertheless, the 14 TPSGMS-based hybrid varieties accounted only 0.5% of total 2691 wheat varieties released in China from 2002 to 2018 [14]. In addition, few hybrid varieties were applied in main producing areas such as Yellow-Huai River wheat zone of China, where inbred varieties perform well in yield while most hybrid varieties available did not exhibit enough yield advantage. An important cause for this situation is that only eight practically usable TPSGMS lines across China were developed over past 20 years, which greatly restricted the opportunities and efficiency of creating heterotic hybrids although ten thousands of restorers were test-crossed. A practical TPSGMS line is commonly characterized as stable sterile duration for ≥20 days in different years [15, 16], out-crossing rate for ≥70% [17], dwarf plant height at 50–70 cm [15], as well as good combining ability and agronomic traits [9]. In addition, the recessive genes controlled sterility only express at a restricted temperature and day-length condition and produces a low ratio of sterile plants in segregating population [15]. These factors result in a low efficiency in developing practical TPSGMS lines by conventional breeding methods. Therefore, the current breeding strategy needs to be improved to develop more practical wheat TPSGMS lines for producing more heterotic hybrids.

The Doubled Haploid (DH) technique allows to homogenize a heterozygous material in one generation. It has been widely used in crop breeding to improve the efficiency of selection and to accelerate the breeding process [18–22]. In wheat, DHs can be produced by intergeneric cross between wheat (*Triticum aestivum* L.) and maize (*Zea mays* L.). This methodology has become an integral part of many commercial wheat breeding programs. It has advantages of stable induction and few genotypic restrictions in producing haploids over anther and microspore cultures [23, 24]. This study aimed to evaluate the efficiency of developing wheat TPSGMS lines by using DH technique based on wheat × maize in breeding program with sterile materials derived from different filial generations of F_1_, F_2_ and F_3_.

## Results

### Successful generation of DH lines by wheat × maize system

**Fig. 1:**
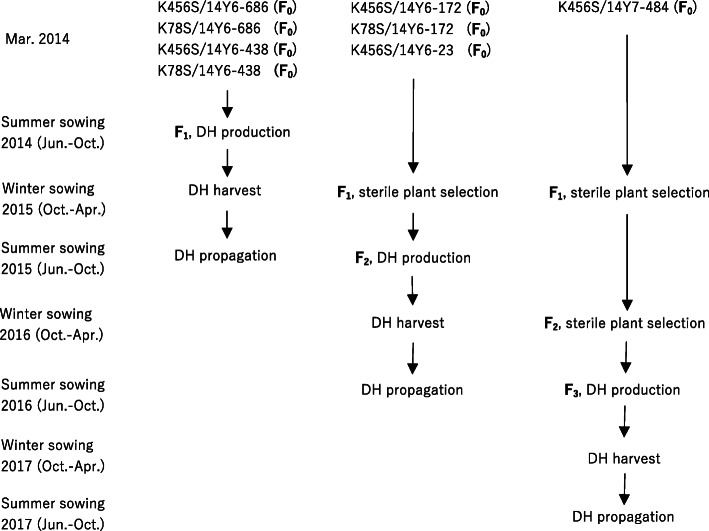
Diagram of making combinations, subsequent breeding work and DH production. Winter sowings of 2015, 2016 and 2017 mean sowings in Oct. of 2014, 2015 and 2016, respectively

During summer sowings in 2014–2016, wheat DHs were produced from four F_1_s and sterile plants selected from F_1_ and F_2_ generations by wheat × maize system (Fig. 1). A total of 920 DH lines were obtained from all eight crosses (Table 1). In Dec. 2016, a major frost caused damage of plants that were heading, resulting in partial failure to obtain DH seeds. Variance analysis showed that there were significant difference in the rate of immature embryos (*P* = 0.00) and haploid seedling rate (No. of seedlings germinated from every 100 inoculated embryos, *P* = 0.00) among different combinations, suggesting that embryo rate and haploid seedling rate were more susceptible to genotypes. The averages of embryo rate, seedling rate of embryos and chromosome doubling rate of seedlings were 36.76, 62.65 and 86.42% respectively, exhibiting a good efficiency in DH production as demonstrated in our previous studies [25–29].

**Table 1:** Parameters of producing doubled haploids with F_1_s and selected sterile plants of F_2_ and F_3_

Year	Combinations	Statistics in producing embryos, haploid seedlings and DH lines^*^							
		NS	NC	NE	NHS	NDH	RE (%)	RHS (%)	RCD (%)
2014/2015	K78S/14Y_6_–686	25	756	136	84	76	17.99^a^	61.76^c^	90.48^cde^
2014/2015	K456S/14Y_6_–686	15	510	214	146	110	41.96^d^	68.22^d^	75.34^a^
2014/2015	K78S/14Y_6_–438	32	928	362	152	130	39.01^c^	41.99^a^	85.53^bc^
2014/2015	K456S/14Y_6_–438	24	654	334	180	148	51.07^g^	53.89^b^	82.22^b^
2015/2016	K78S/14Y_6_–172	16	564	152	110	102	26.99^b^	72.37^e^	92.73^e^
2015/2016	K456S/14Y_6_–172	18	532	234	147	128	43.98^e^	62.82^c^	87.07^bcd^
2015/2016	K456S/14Y_6_–23	11	360	173	130	119	48.06^f^	75.14^e^	91.54^de^
2016/2017	K456S/14Y_7_–484	40	1292	323	210	107	25.00^b^	65.02^cd^	–^**^
Total / average		181	5596	1928	1159	920	36.76	62.65	86.42

^*^NS No. of pollinated spikes, *NC* No. of caryopses, *NE* No. of embryos obtained, *NHS* No. of haploid seedlings germinated from embryos, *NDH* No. of DHs, *RE* Rate of embryo (NE·NC^− 1^), *RHS* Rate of haploid seedling (NHS·NE^− 1^), *RCD* Rate of chromosome doubling (NDH·NHS^− 1^)

^**^Part of colchicine treated plants was damaged by frost. Different letters in the last three rows mean significant at 0.05 level

**Fig. 2:**
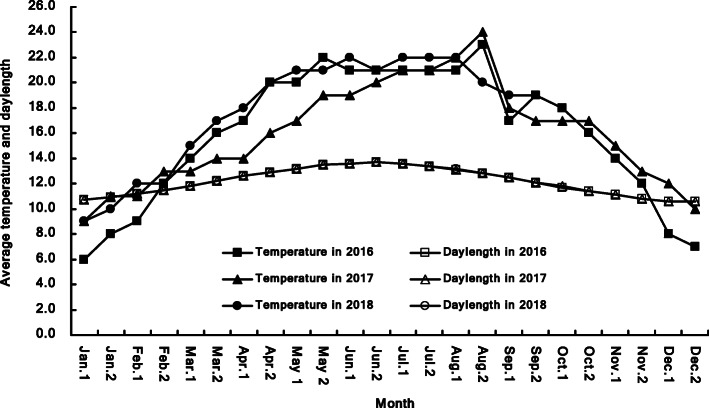
Average temperature and daylength of every half month from 2016 to 2018 at Kunming. Jan.1 and Jan.2 mean the first and second half of Jan., the same as that of other months; data were collected from Jan. 1, 2016 to Sep. 30, 2018

Temperate climate at Kunming, especially from May to October, allows planting spring and vernalized winter wheat materials throughout the year under natural condition (Fig. 2 and Supplemental Data 1), which facilitates mass production of wheat DHs by wheat × maize crosses because fresh pollens are available from multiple rounds of planting of maize plants from late April to early November [27, 29].

### Candidates of elite DH sterile lines

**Fig. 3:**
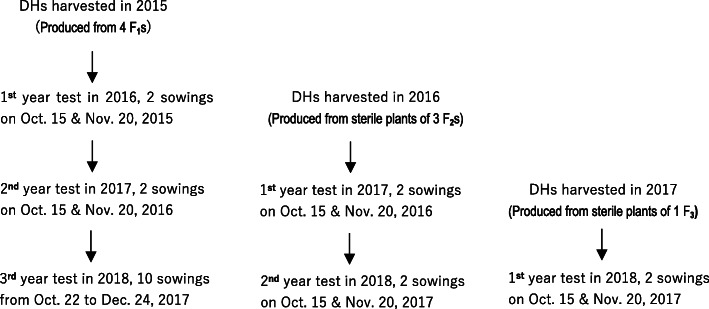
Diagram of sterility tests for DHs produced from F_1_, F_2_ and F_3_ generations. Each sowing in the 3rd year test was conducted at 7 days interval from Oct. 22 to Dec. 24, 2017

**Fig. 4:**
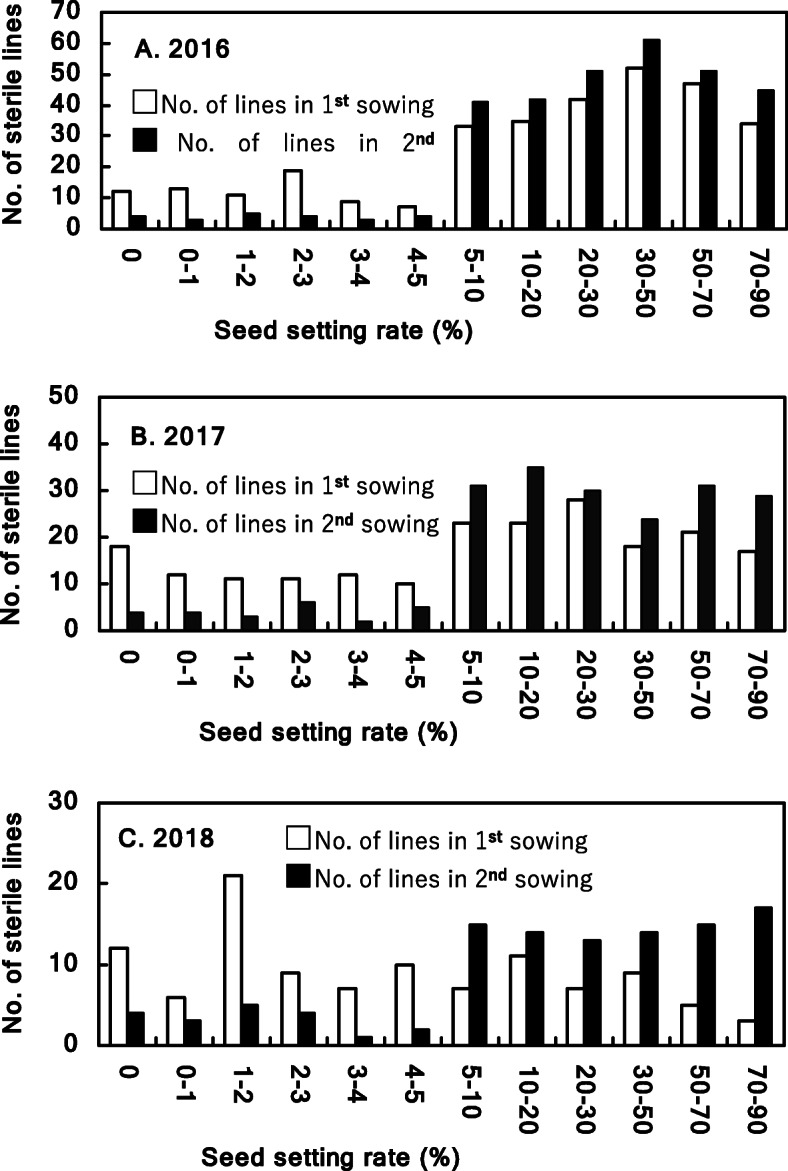
Distribution of seed setting rate of 625 DHs in first year sterility tests during 2016–2018. 314 DH lines were tested in 2016 (**a**), 204 DH lines in 2017 (**b**), and 107 DH lines in 2018; the 1st sowing dates were on Oct. 15 in 2015 (**a**), 2016 (**b**) and 2017 (**c**), respectively; the 2nd sowing dates were on Nov. 20 in 2015 (**a**), 2016 (**b**) and 2017 (**c**), respectively

During 2016–2018, all 920 DH lines produced from F_1_, F_2_ and F_3_ generations were evaluated independently using a one-year sterility test to screen candidate sterile lines (Fig. 3). A total of 295 DH lines showed normal seed set were excluded from further analysis. These lines were mostly from F_1_ generations as expected. In the 1st (Oct. 15) and 2nd (Nov. 20) sowings, 210 (33.60%) and 66 (10.65%) lines from the total of 625 DH lines had seed setting rates less than 5%, respectively. Furthermore, 41 (6.56%) DH lines showed seed setting rate less than 5% in both sowings (Table 2, Fig. 4 and Supplemental Data 2). When further considering other desired traits of out-crossing potential, disease resistance (to stripe rust and powdery mildew), plant height (50–60 cm), tillering and spike formation ability, 24 lines were selected from 41 lines as our final set of elite DH lines*.*

**Table 2:** Results of sterile lines selection in sterility tests of 2015/2016, 2016/2017 and 2017/2018 seasons

Year	Source of DH lines	No. of tested lines	No. of lines with seed setting rate < 5%			Finally selected elite lines	
			1st sowing	2nd sowing	Both sowings^a^	Number	%
2015/2016	F_1_	314	71	23	13 (4.14 C)	10	3.18 C
2016/2017	F_2_	204	74	24	15 (7.35 B)	10	4.90 A
2017/2018	F_3_	107	65	19	13 (12.15 A)	4	3.74 B
Total		625	210	66	41 (6.56)	24	3.84

^a^Figures in parentheses denote the corresponding percentages in yearly tested lines. Different letters in the sixth and eighth rows mean significant at 0.01 level by U-test

When sterile lines are sown on Oct. 15 (1st sowing) and Nov. 20 (2nd sowing), the critical periods for causing fertility alteration are from middle to late February and from late March to early April, respectively. Consequently, during the critical periods, the 1st sown sterile lines would go through lower temperature and shorter days to fully exhibit sterility, while the 2nd sown lines would have relatively higher temperature and longer days that can cause the early heading spikes sterile and the late heading tillers partially fertile to produce a few seeds for propagation (Fig. 2 and Supplemental Data 1).

Based on our experience, TPSGMS lines that exhibit 100% sterility in the 2nd sowing date are usually stable in sterility but are difficult for propagation, which make them not suitable for practical application. In southwest of China wheat is normally sown from middle Oct. to early Nov., thus a TPSGMS line with seed setting rate < 5% in both sowing dates (from Oct. 15 to Nov. 20) would meet the demand for safe production of qualified hybrid seeds.

### F_1_-derived elite sterile lines exhibited stable sterility

**Fig. 5:**
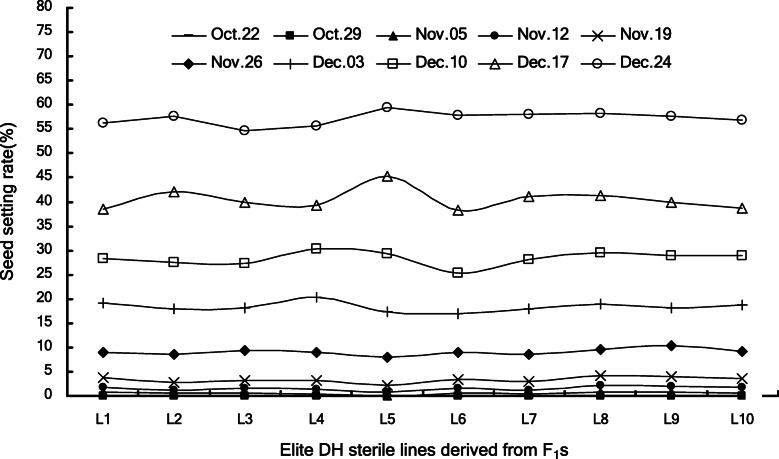
Seed setting rates of ten elite TPSGMS lines in ten sowings of 2017/2018 growing season. Sowing dates started from Oct. 22, 2017 with an interval of seven days

To test the stability of sterility in different years, ten F_1_-derived elite DH sterile lines were evaluated in two growing seasons from 2016 to 2018 by planting in two and ten different sowing dates respectively (Fig. 3). In the 2016/2017 growing season, the seed setting rates of all lines were 0 in the 1st sowing date, and ranged from 2.98 to 4.87% in the 2nd sowing date (Table 3). In further tests using ten sowing dates in the 2017/2018 season, the seed setting rates of the ten elite lines were < 1% from the 1st to the 3rd sowings (Oct.22-Nov. 5), < 5% till the 5th sowing (Nov. 19), and ≥ 50% in the 10th sowing (Dec. 24), suggesting sowings before Nov. 5–19 were optimum for hybrid seed production, and sowings after Dec. 24 is suitable for propagation of these sterile lines (Fig. 5 and Supplemental Data 3).

**Table 3:** Seed setting rates of F_1_ derived elite lines in second round sterility test in 2017

Tested code	L1	L2	L3	L4	L5	L6	L7	L8	L9	L10	CK
Line code	16DH002	16DH005	16DH014	16DH087	16DH102	16DH106	16DH203	16DH256	16DH284	16DH303	K78S
1st sowing^a^	0.00	0.00	0.00	0.00	0.00	0.00	0.00	0.00	0.00	0.00	0.00
2nd sowing^b^	4.72	3.76	4.23	4.21	2.98	4.42	3.79	4.87	4.81	4.46	4.85

^a^Sowing on Oct. 15, 2016, ^b^sowing on Nov. 20, 2016

The ten elite TPSGMS lines derived from F_1_ generation showed nearly 100% sterility in 3 years when sown from Oct. 15 to Nov. 5, although the average temperatures varied from 12 °C to 15 °C during the critical periods from the second half Feb. to the first half Mar. (Fig. 2 and Supplemental Data 1). These lines are stable in sterility in 3 years’ tests, thus selected for hybrid breeding. Stable sterility is a prerequisite to commercial utilization for a TPSGMS line [16].

### Sterility stability of F_2_-derived elite sterile lines was confirmed by repeat tests

Ten F_2_-derived DH lines were selected in 2016/2017 and were evaluated again in the 2017/2018 growing season (Fig. 3). The seed setting rates of all lines were 0 in the 1st sowing, and ranged from 1.99 to 4.04% in the 2nd sowing (Table 4). These ten lines showed stable sterility in 2 years, were selected to plant in ten sowing dates for further evaluation in stability of sterility, as well as determination of suitable sowing times for hybrid seed production and self propagation.

**Table 4:** Seed setting rates of F_2_ derived elite lines in second round sterility test in 2018

Tested lines	17DH007	17DH013	17DH026	17DH039	17DH065	17DH104	17DH157	17DH158	17DH192	17DH202	CK
1st sowing^a^	0.00	0.00	0.00	0.00	0.00	0.00	0.00	0.00	0.00	0.00	0.00
2nd sowing^b^	3.76	2.76	1.99	3.79	3.72	4.04	2.38	3.24	3.17	3.92	4.09

^a^Sowing on Oct. 15, 2017, ^b^sowing on Nov. 20, 2017; CK = K78S

### Elite DH sterile lines showed high out-crossing ability

In winter sowing of 2018/2019, the out-crossing potential of 20 elite TPSGMS lines derived from F_1_ and F_2_ generations were evaluated. The out-crossing rates of the 20 lines ranged from 70.46 to 93.90% with an average of 82.87%. There were 13 lines, including 8 derived from F_2_ generation, showing out-crossing rate > 80%, 4 lines between 75 and 80%, and 3 lines between 70 and 75% (Table 5). All 20 lines showed high out-crossing potential after only one round of selection after DH production. Thus, doubled haploids showed a great efficiency in fixation of this trait, which confirmed our previous results [17]. More lines derived from F_2_ generation appeared to have better out-crossing ability (> 80%) compared with those from F_1_ generation, suggesting that one more cycle of selection before DH production is helpful to further enhance the target trait. The results of out-crossing rates here were obtained by pollination with nearly unlimited pollen supply, it needs to be further confirmed in practical hybrid seed production.

**Table 5:** Out-crossing rates of 20 elite DH sterile lines derived from F_1_ and F_2_ in 2019^a^

Lines	Source	ugn	bgn	SN	OR	Lines	Source	ugn	bgn	SN	OR
17D007	F_2_	59.50	0.00	40.40	73.64	16D002	F_1_	55.20	0.10	39.10	70.46
17D013	F_2_	65.20	0.05	38.70	84.17	16D005	F_1_	70.30	0.00	40.20	87.44
17D026	F_2_	69.70	0.00	39.60	88.01	16D014	F_1_	72.50	0.00	41.00	88.41
17D039	F_2_	60.50	0.10	38.80	77.84	16D087	F_1_	65.40	0.00	43.40	75.35
17D065	F_2_	70.40	0.00	38.80	90.72	16D102	F_1_	61.50	0.10	36.30	84.57
17D104	F_2_	69.70	0.05	40.00	87.06	16D106	F_1_	60.20	0.00	38.60	77.98
17D157	F_2_	78.50	0.00	41.80	93.90	16D203	F_1_	72.50	0.00	41.90	86.52
17D158	F_2_	76.30	0.05	45.10	84.53	16DH256	F_1_	68.40	0.00	45.90	74.51
17D192	F_2_	74.50	0.00	43.60	85.44	16DH284	F_1_	74.80	0.10	43.30	86.26
17D202	F_2_	72.50	0.00	42.80	84.70	16DH303	F_1_	57.80	0.00	38.10	75.85
Average		69.68	0.03	40.96	85.00	Average		65.62	0.03	40.78	80.73

^a^All sterile lines were sown on Oct. 15, 2018. ugn and bgn represent No. of seeds in un-bagged and bagged spikes, SN denotes No. of spikelets per spike, OR means the out-crossing rate

### Comparison of DH breeding efficiency among generations

Based on the seed setting rates < 5% in both sowing dates, 41 DH sterile lines, including 13, 15, and 13 lines derived from F_1_, F_2_ and F_3_ generations, respectively, were selected (Table 2), the breeding efficiency (percentage of selected DH lines in total DH lines tested) was 4.14, 7.35 and 12.15% for F_1,_ F_2_ and F_3_, respectively. A total of 24 elite lines were selected based on further evaluation of other desired traits. The breeding efficiency for F_1,_ F_2_ and F_3_ was 3.18, 4.90 and 3.74%, respectively. U-test analyses indicated that there were significant differences (*P* < 0.01) in breeding efficiency of producing DHs from F_1_, F_2_ and F_3_ generations (Table 2). The trend of breeding efficiency for a single trait (sterility) was in the order of F_3_ > F_2_ > F_1_, while for comprehensive traits was F_2_ > F_3_ > F_1_, which suggests that producing DHs from F_2_ generation is better in overall breeding efficiency.

## Discussion

Discovery and application of male sterility is the foundation of commercial production of hybrid wheat. The chance of creating elite heterotic hybrids is correlated with the number of sterile lines and restorers available in breeding programs. Although the TPSGMS-based two-line hybrid wheat system was established in 1990s [7, 30], less than ten TPSGMS lines capable for commercial usage have been developed in north and south wheat zones of China up to now. Pedigree method is commonly used in developing TPSGMS lines [7, 15, 30, 31], however, several difficulties have hindered the breeding efficiency. The sterility of TPSGMS line is controlled by two or three recessive major genes plus several minor genes [7, 16, 32–34], causing a very low proportion of highly sterile plants in segregating populations, especially in F_2_s derived from crosses between sterile lines and normal fertile lines. When other desired traits are considered together during selection, the breeding efficiency would become extremely low. Theoretically, the probability of homozygous recessive individuals in F_2_ population is 1/4^n^, the probability would be 1/2^n^ if DH lines are produced from F_1_, suggesting DH breeding is more efficient for selection of traits controlled by recessive genes, such as the sterility here. Crosses between semi-sterile materials and sterile lines can further increase the proportion of highly sterile plants in segregating populations of this study, which is similar in effectiveness to backcrossing with sterile lines [31].

Few effective molecular markers are currently available for marker-assisted selection in sterility of wheat TPSGMS lines [35]. Consequently, it is time-consuming to develop a genetically stable TPSGMS line because the expression of sterility needs strict temperature and light condition which is only available one season per year [15]. In our previous breeding program, only two practical TPSGMS lines (K78S and K456S) were developed by pedigree method from 1996 to 2010, while in this study we developed 24 elite TPSGMS lines with complete homozygosity and other desired traits within 4 years by introducing DH techniques.

Another issue addressed in this study is to identify the ideal generation for producing DHs. Most breeders prefer to produce DHs from F_1_ generation to shorten the breeding cycles, but this approach may limit the chances for recombination [18]. Therefore, producing DHs with selected individuals from F_2_ generation of single crosses or F_1_ generation of pyramiding crosses seems to be better than that from F_1_ generation of single crosses [36]. Similarly, Snape and Simpson (1981) inclined to produce DHs from F_2_ generation in barley by comparing the gain in genetic variation for six agronomic traits with DH lines derived from F_1_, F_2_, F_3_ and intermated F_2_ (S3) generations [37]. In contrast, Iyamabo and Hayes (1995) did not find more favorable genotypes in DH lines produced from F_2_ generation than that from F_1_ generation in barley, therefore, they preferred to use F_1_ generation for producing DHs [38]. In the present study, the overall breeding efficiency of producing DHs from filial generations was in the order of F_2_ > F_3_ > F_1_, indicating that F_2_ generation is better for producing DHs in breeding efficiency. However, it still needs to be further investigated by comparing the breeding efficiency of producing DHs with F_1_ and selected plants of F_2_ and F_3_ derived from the same cross.

Producing DHs from F_1_ generation had less breeding efficiency because only one round of recombination occurred and no selection was applied. As a result, a high frequency of agronomically undesirable lines were produced [37], which was confirmed in this study as most fertile lines were discarded from F_1_ generation. However, as showed in this study, using F_1_ generation for DH breeding has the edge in saving time; it could be useful for crosses with better predictability and coping with urgent needs for developing varieties with resistance to diseases, such as yellow rust for its frequently varying pathogenic races.

## Conclusion

In this study we developed 20 practical TPSGMS lines of wheat. These lines showed stable sterility in replicated evaluation in multiple years, as well as good outcrossing potential and other desirable traits. We demonstrated that introducing DH technique is an efficient strategy in accelerating development of TPSGMS lines of wheat. Producing DHs from F_2_ generation appeared to be the better choice with balance of breeding efficiency and shortening of breeding cycle. Nevertheless, it will be necessary to conduct further investigations by using diverse genetic materials of different filial generations derived from the same combinations. More practically usable TPSGMS lines would further increase the opportunity of creating heterotic hybrids in hybrid wheat breeding.

## Methods

### Plant materials

Two TPSGMS lines and five semi-sterile advanced lines of wheat were used in the study (Table 6). A maize variety “Baitiannuo” was used as pollen donor in DH production. All wheat and maize materials were bred by Institute of Food Crops, Yunnan Academy of Agricultural Sciences, Kunming, China.

**Table 6:** wheat materials used for developing TPSGMS lines

Materials	Description
K78S, K456S	Commercial TPSGMS lines, susceptible to stripe rust and powdery mildew, different in yield potential and sterility, used as female parents.
14Y6–686, 14Y6–438, 14Y6–23, 14Y6–172, 14Y7–484	Semi-sterile advanced lines, resistant to stripe rust and powdery mildew, different in plant height, heading time and yield potential, used as male parents.

### Crossing and DH production

Wheat materials were late sown in Jan. 2014 to make semi-sterile materials fertile for crossing with sterile lines K78S and K456S in Mar. 2014 at Kunming, Yunnan province, China (25°02′N, 102°42′E, altitude 1960 m). For DH production, maize sowing (in April) began 2 months before wheat sowing (in June) to synchronize their flowering dates. Maize was sown in three dates with an interval of 14 days.

Before producing doubled haploids, pedigree methods were adopted to select sterile plants from segregating population of F_1_ and F_2_ generations according to performances in sterility, out-crossing potential including glume opening and stigma exsertion [17], plant height (50–60 cm), resistance to yellow rust and powdery mildew, tillering ability and the yield potential. Seeds of sterile plants were harvested from regenerated tillers by cutting all spikes of sterile plants followed by intensive management in irrigation and fertilization. The crossings and subsequent breeding work and DH production are summarized in Fig. 1.

### Method of producing DHs

For DH production, we adopted an improved protocol from previous reports [18, 23, 24]. Wheat spikes were pollinated with fresh maize pollen 24 to 48 h after emasculation. Pollinated tillers were cut 24 h after pollination and sprayed with 100 ppm 2,4-D, then were cultured in growth chambers for 14 days with nutrition solution containing 100 mg L^− 1^ 2,4-D, 40 g L^− 1^ sucrose, 10 mg L^− 1^ silver nitrate, 3 g L^− 1^ potassium dihydrogen phosphate and 3 g L^− 1^ urea. The nutrient solution was replaced every 3 days. During culture, the growth chamber keeps a regime of 14 h darkness / 10 h light with light intensity of 6000 Lux, constant temperature of 25 ± 1 °C and relative humidity of 80 ± 5%. Embryos were aseptically dissected from 14-day caryopses and cultured on half-strength MS medium [39] under darkness at 24 ± 1 °C until germination, then moved to the growth house at a regime of 14 h darkness /10 h light (at 3000 Lux), keeping constant temperature of 25 ± 1 °C and humidity of 75 ± 5%. When seedlings developed two to three tillers, the plantlets were taken out and immersed in 0.05% colchicine solution for 8 h at 25 °C to induce doubling of chromosomes. Treated seedlings were transplanted into pots to grow until booting stage, then moved into greenhouse for 15 days, keeping temperature > 20 °C to ensure fertility of DH plants. All plants were bagged before flowering and harvested one by one.

### DH sterile line selection and its stability evaluation in sterility

The sterility of DH lines obtained during 2015–2017 was independently evaluated by sowing at two dates on Oct.15 and Nov. 20, respectively (Fig. 3). At least 10 spikes per line in each sowing were randomly bagged before flowering to measure the seed setting rate. The out-crossing potential (glume opening, stigma exsertion) [17], and other important traits such as disease resistances and yield potential were also recorded. Lines with sterility higher than 95% in both sowings were kept for stability evaluation of sterility next year.

Selected lines in 2015/2016 and 2016/2017 growing seasons were repeatedly evaluated in 2016/2017 and 2017/2018 seasons. For F_1_-derived elite DH lines, we also conducted a ten-sowing assessment in the third year from Oct. 22, 2017 to Dec. 24, 2017 with an interval of 7 days. The TPSGMS line K78S was used as the check in all tests. The seed setting rate (SSR) was calculated following Yang et al. (2006) [16]:

$$ SSR\ \left(\%\right)= gn/\left( sn\times 2\right)\times 100 $$

Where *gn* means the number of grains from bagged spikes, *sn* the number of spikelets. A TPSGMS line with SSR < 5% was recognized as highly sterile and qualified for hybrid seed production [16].

Temperatures during 2016–2018 were collected from a data-logger ‘HUATO S100-TH’ in thermometer screen near the field, and daylengths from the meteorological station of Kunming.

### Out-crossing potential assessment of elite sterile lines

Twenty elite DH sterile lines derived from F_1_ and F_2_ generations were separately planted as 10 rows in a plot of 1 m × 2.5 m surrounded by about 600 restorers on Oct. 15, 2018. Open pollination was aided by natural wind of grade 3–6, which is usual in Yunnan throughout wheat growing seasons. Twenty spikes of each line were randomly bagged before flowering. Twenty open pollinated spikes were randomly harvested from 10 rows of each line with the bagged spikes to measure the out-crossing rate (OR) of sterile lines as follow [16, 17]:

$$ OR\ \left(\%\right)=\left( ugn- bgn\right)/\left( sn\times 2\right)\times 100 $$

Where *ugn* and *bgn* are un-bagged and bagged grain numbers at two basal florets of each spikelet respectively, *sn* the number of spikelets per spike. *bgn* is counted to exclude the possible self-pollination seed setting because TPSGMS lines are not always keeping 100% sterile.

### Evaluation of disease resistance

Yellow rust and powdery mildew are two most important and frequently occurred diseases at Kunming. A highly susceptible cultivar was planted close to tested DH sterile lines as the control and spreader. The adult plant resistance of sterile lines was scored in the field according to Han et al. (2010) for yellow rust [40] and Li et al. (2015) for powdery mildew [41].

### Statistical analysis

The embryo rate, haploid seedling rate and chromosome doubling rate were analyzed using one-way analysis of variance (ANOVA) followed by Fisher’s least significant difference (LSD) test. U-test of multiple percentage comparison [42] was conducted for evaluating the significant differences in breeding efficiency of producing DHs from F_1_, F_2_ and F_3_ generations. SPSS and Excel Office were used in statistical analyses.

## Supplementary information

**Additional file 1: Supplemental Data 1.** Raw data for Fig. 2: The temperature and daylength during 2016–2018.

**Additional file 2: Supplemental Data 2.** Raw data for Fig. 4: Seed setting rates in the first year’s sterility test of 625 DH lines in both sowings during 2016–2018.

**Additional file 3: Supplemental Data 3.** Raw data for Fig. 5: The seed setting rates of ten elite lines in ten sowings of 2017/2018 season.

## Data Availability

Plant lines and data generated in this study are available upon reasonable request, from the corresponding author.

## Abbreviations

TPSGMS: Thermo-photo sensitive genic male sterility
DH: Doubled haploid
NS: Number of pollinated spikes
NC: Number of caryopses
NE: Number of embryos
NHS: Number of haploid seedlings germinated from embryos
NDH: Number of double haploids
RE: Rate of embryos
RHS: Rate of haploid seedling
RCD: Rate of chromosome doubling
SSR: Seed setting rate
gn: grain number of bagged spikes
sn: spikelet number
OR: Out-crossing rate
ugn: un-bagged grain numbers at two basal florets of each spikelet
bgn: bagged grain numbers at two basal florets of each spikelet
